# CLIC-01: Manufacture and distribution of non-cryopreserved CAR-T cells for patients with CD19 positive hematologic malignancies

**DOI:** 10.3389/fimmu.2022.1074740

**Published:** 2022-12-19

**Authors:** Natasha Kekre, Kevin A. Hay, John R. Webb, Ranjeeta Mallick, Miruna Balasundaram, Mhairi K. Sigrist, Anne-Marie Clement, Julie S. Nielsen, Jennifer Quizi, Eric Yung, Scott D. Brown, Lisa Dreolini, Daniel D. Waller, Julian Smazynski, Nicole S. Gierc, Bianca C. Loveless, Kayla Clark, Tyler Dyer, Richard Hogg, Leah McCormick, Michael Gignac, Shanti Bell, D. Maria Chapman, David Bond, Siao Yong, Rachel Fung, Heather M. Lockyer, Victoria Hodgson, Catherine Murphy, Ana Subramanian, Evelyn Wiebe, Piriya Yoganathan, Liana Medynski, Dominique C. Vaillan, Alice Black, Sheryl McDiarmid, Michael Kennah, Linda Hamelin, Kevin Song, Sujaatha Narayanan, Judith A. Rodrigo, Stefany Dupont, Terry Hawrysh, Justin Presseau, Kednapa Thavorn, Manoj M. Lalu, Dean A. Fergusson, John C. Bell, Harold Atkins, Brad H. Nelson, Robert A. Holt

**Affiliations:** ^1^ Clinical Epidemiology Program, Ottawa Hospital Research Institute, Ottawa, ON, Canada; ^2^ Division of Hematology, Department of Medicine, The Ottawa Hospital, Ottawa, ON, Canada; ^3^ Division of Hematology, Department of Medicine, University of British Columbia, Vancouver, BC, Canada; ^4^ Terry Fox Laboratory, British Columbia Cancer Research Institute, Vancouver, BC, Canada; ^5^ Vancouver General Hospital, Leukemia and Bone Marrow Transplant Program of British Columbia, Vancouver, BC, Canada; ^6^ Conconi Family Immunotherapy Lab, Trev and Joyce Deeley Research Centre, British Columbia Cancer Research Institute, Victoria, BC, Canada; ^7^ Canada’s Michael Smith Genome Sciences Centre, British Columbia Cancer Research Institute, Vancouver, BC, Canada; ^8^ Center for Innovative Cancer Therapeutics, Ottawa Hospital Research Institute, Ottawa, ON, Canada; ^9^ Department of Biochemistry, Microbiology and Immunology, University of Ottawa, Ottawa, ON, Canada; ^10^ School of Epidemiology and Public Health, University of Ottawa, Ottawa, ON, Canada; ^11^ Department of Cellular Molecular Medicine, University of Ottawa, Ottawa, ON, Canada; ^12^ Department of Biochemistry and Microbiology, University of Victoria, Victoria, BC, Canada; ^13^ Department of Medical Genetics, University of British Columbia, Vancouver, BC, Canada; ^14^ Department of Molecular Biology & Biochemistry, Simon Fraser University, Burnaby, BC, Canada

**Keywords:** CAR-T cells, hematologic malignancies, immune effector cells, point-of-care manufacturing, prodigy, in-house manufacturing, lymphoma, leukaemia

## Abstract

Access to commercial CD19 CAR-T cells remains limited even in wealthy countries like Canada due to clinical, logistical, and financial barriers related to centrally manufactured products. We created a non-commercial academic platform for end-to-end manufacturing of CAR-T cells within Canada’s publicly funded healthcare system. We report initial results from a single-arm, open-label study to determine the safety and efficacy of in-house manufactured CD19 CAR-T cells (entitled CLIC-1901) in participants with relapsed/refractory CD19 positive hematologic malignancies. Using a GMP compliant semi-automated, closed process on the Miltenyi Prodigy, T cells were transduced with lentiviral vector bearing a 4-1BB anti-CD19 CAR transgene and expanded. Participants underwent lymphodepletion with fludarabine and cyclophosphamide, followed by infusion of non-cryopreserved CAR-T cells. Thirty participants with non-Hodgkin’s lymphoma (n=25) or acute lymphoblastic leukemia (n=5) were infused with CLIC-1901: 21 males (70%), median age 66 (range 18-75). Time from enrollment to CLIC-1901 infusion was a median of 20 days (range 15-48). The median CLIC-1901 dose infused was 2.3 × 10^6^ CAR-T cells/kg (range 0.13-3.6 × 10^6^/kg). Toxicity included ≥ grade 3 cytokine release syndrome (n=2) and neurotoxicity (n=1). Median follow-up was 6.5 months. Overall response rate at day 28 was 76.7%. Median progression-free and overall survival was 6 months (95%CI 3-not estimable) and 11 months (95% 6.6-not estimable), respectively. This is the first trial of in-house manufactured CAR-T cells in Canada and demonstrates that administering fresh CLIC-1901 product is fast, safe, and efficacious. Our experience may provide helpful guidance for other jurisdictions seeking to create feasible and sustainable CAR-T cell programs in research-oriented yet resource-constrained settings.

**Clinical trial registration:**

https://clinicaltrials.gov/ct2/show/NCT03765177, identifier NCT03765177.

## Introduction

Chimeric Antigen Receptor (CAR)-T cells are a powerful tool for treating cancer. CAR-T cells are genetically modified T cells that are programmed to target a specific cell surface antigen. The use of CAR-T cells has been explored in clinical trials for various cancers, mainly hematologic malignancies such as B-cell acute lymphoblastic leukemia (1) and non-Hodgkin’s lymphoma (2–4), where the pooled complete response rate is 56% (95% CI: 44%, 67%; I2 69%) (5). This degree of efficacy is remarkable given that most of these patients only had palliative options prior to CAR-T therapy and has prompted rapid regulatory approval of CD19 CAR-T cell interventions for these cancers worldwide (6–8).

Despite this promise, CAR-T cell production and distribution is complex and operationally challenging, which hampers access to these lifesaving therapies. At the time of writing, Canadian cancer patients have highly variable access to CAR-T cell therapy depending on jurisdiction. While most CAR-T products are currently manufactured in centralized facilities, academic CAR-T studies have explored various approaches to in-house manufacturing and delivery of CAR-T cells, and in clinical trials these efforts have yielded high quality products with safety and efficacy profiles that compare favourably to commercially available CAR-T cell products (9–14). This paradigm has been greatly facilitated by closed benchtop systems that make manufacturing within the hospital setting more feasible. Miltenyi’s CliniMACS Prodigy is one such closed system in which the patient’s T cells, CAR-encoding virus, cytokines and T-cell growth media are all manipulated on the instrument within a single-use closed tubing set. The Prodigy has a relatively small footprint, allowing multiple instruments to be operated in a single facility. Notable advantages to in-house CAR-T manufacturing include opportunities to actively manage production capacity, pursue continuous process improvement, minimize the cost of these expensive interventions (15), and ultimately improve their value for money.

Our research consortium was established to test the feasibility of providing high quality, cost effective, in-house manufactured CD19 CAR-T cells within Canada’s financially strained healthcare system. We designed, manufactured, and functionally verified a “2nd generation” 4-1BB-containing CD19 CAR construct and lentiviral vector system to enable CAR-T manufacturing using the Miltenyi Prodigy platform. All aspects of vector and CAR-T manufacturing, clinical trial design, patient enrollment and treatment, clinical trial monitoring, and regulatory filings were undertaken by our academic team.

Here we report interim results from CLIC-01, a single-arm, open-label phase I/II study (NCT03765177) to evaluate the safety and efficacy of our CLIC-1901 non-cryopreserved CAR-T cell product in participants with CD19 positive hematologic malignancies. We successfully implemented this trial at two Canadian hospitals on opposite sides of the country, with cell manufacturing at a third site. We leveraged hematopoietic stem cell transplantation protocols already available in Canada to successfully transport fresh apheresis and final CAR-T products, with no delays or interruptions despite our large geography. In fact, the time sensitivity of transporting rapidly expiring cell products over large distances imparted a sense of urgency to this study, which benefitted patients by removing any possibility of delay when treating their rapidly progressing malignancies. This clinical trial represents an important step towards enhancing CAR-T cell innovation and equitable access for Canadian cancer patients and may offer helpful guidance to other jurisdictions seeking to incorporate this transformative form of cancer treatment in resource-constrained settings.

## Methods

### Study protocol

The protocol and amendments were reviewed by Health Canada prior to implementation and approved by the institutional review boards at both study sites, Vancouver General Hospital (VGH) and The Ottawa Hospital (TOH). Participants aged 18 years or older were eligible if they had relapsed or refractory CD19 positive disease including acute lymphoblastic leukemia (ALL), chronic lymphocytic leukemia (CLL) or histologically confirmed B-cell non-Hodgkin’s lymphoma (NHL). Relapsed or refractory disease was defined by one of the following: a) second or greater relapse, b) any relapse after autologous or allogeneic stem cell transplantation, or c) chemorefractory as defined by not achieving complete remission after 2 cycles of a standard induction chemotherapy or 1 cycle of salvage therapy. All eligible participants had to have documentation of CD19 tumour expression demonstrated in tissue biopsy, bone marrow or peripheral blood within 6 months prior to study screening, as well as adequate organ function defined as: creatinine clearance > 30 mL/min, ALT/AST < 3X upper limit of normal (ULN), and bilirubin < 2X ULN. Exclusion criteria included: isolated extra-medullary disease, concomitant genetic syndrome (such as Fanconi anemia, or any other known familial bone marrow failure syndrome), malignancy in the last 5 years or concurrent active malignancy (with the exception of non-melanomatous skin cancer), prior treatment with any gene therapy product, PCR positive hepatitis B, C or HIV, any uncontrolled infection, active graft-versus-host disease requiring systemic therapy, allogeneic stem cell transplant less than 6 months prior to CLIC-1901 cell infusion or donor lymphocyte infusion less than 6 weeks prior to CLIC-1901 cell infusion, active Central Nervous System (CNS) involvement by malignancy, history of anaphylaxis to gentamicin or its derivatives, or participants receiving an investigational agent within the 30 days prior to enrolment.

### Lentivector production

The **Supplementary Material** provides detailed methods for lentivector and CAR-T manufacturing (S1). The CLIC-1901 CAR construct is a second-generation CAR as described by Imai et al, 2004 (16), with modifications to the leader and linker sequences, as described by Kochenderfer et al. (17). In brief, the CAR consists of a GM-CSF receptor alpha (GM-CSFRα) signal peptide, the scFv fragment derived from the FMC63 mouse monoclonal antibody, the CD8α-derived hinge and transmembrane region, a 4-1BB co-stimulatory domain, and a CD3ζ signaling domain. We created a three plasmid self-inactivating lentivector system (18, 19) comprising a packaging plasmid encoding gag, pol and rev, a VSVG envelope protein expressing plasmid (VSV-G), and a transfer plasmid encoding the CAR transgene. The design incorporates standard biosafety features (20). Synthetic transgene sequences encoding the CLIC-1901 CAR were manufactured, subcloned into the transfer plasmid and verified by Sanger sequencing. Clinical grade plasmid production of all DNA was carried out at BC Cancer Research Institute (Vancouver, Canada) using endotoxin-free Purelink Expi Giga Plasmid Purification Kit (Invitrogen #A31232).

Plasmid DNA was used by the Biotherapeutics Manufacturing Centre (BMC) at Ottawa Hospital Research Institute (OHRI) to create replication-incompetent CD19-CAR lentivirus by transient transfection of a master cell bank of 293T/17 cells (ATCC CRL-11268) and expansion in 36-layer HYPERstacks (Corning). Crude supernatant was clarified, treated with benzonase to remove residual cellular DNA, and concentrated *via* tangential flow filtration (TFF). Diafiltration was performed to formulate the CD19 CAR lentivirus in TexMACS Medium (Miltenyi), further concentrated using high-speed centrifugation, resuspended in TexMACS buffer (Miltenyi) and then aliquoted into 2mL cryovials at 500 µL/vial and stored at -80°C. The following release tests were performed on the final *CD19CAR* lentiviral product: 1) host cell DNA detection by qPCR, 2) residual benzonase quantitation, 3) residual VSV-G plasmid, E1A, or SV40 detection, 4) endotoxin level, 5) sterility, 6) pH & appearance, 7) identity, and 8) titer assay. Additionally, the replication-competent lentivirus release test was performed on the crude lentivirus harvest, and four release tests (for mycoplasma, 9 CFR Bovine Virus, adventitious viral contaminants, and replication competent lentivirus) were performed on the control cells in spent media. Empirical evaluation of the percentage of CAR-T cells generated using the Miltenyi Prodigy protocol per volume of lentivirus provided indicated that one vial per manufacturing run was favourable in terms of handling and transduction efficiency.

### CAR-T cell production

Patients underwent apheresis using a standard MNC collection procedure. Apheresis volume ranged from 219 to 348 mL. Immediately following apheresis collection, the product was packaged in a standard transfer device (Credo cube) and shipped cold (1-10°C), but without cryopreservation, from the apheresis location (Ottawa, Ontario or Vancouver, British Columbia) to the cell processing facility in Victoria, British Columbia. CAR-T cells were manufactured using the Miltenyi CliniMACS Prodigy system installed in a classified Grade D manufacturing suite. The exception to this is that the first four manufacturing runs used apheresis that was shipped at ambient temperature (15-25°C). ‘Functionally open’ steps (i.e., media preparation and cell manipulation) were performed in a classified Grade A isolator (NuAire). We followed the ‘enhanced feeding protocol’ version of the pre-installed T cell transduction (TcT) protocol for serum-free cultivation. In-process control samples obtained on day 5 and final product samples obtained on day 12 were subjected to sterility testing using a BacT ALERT system (BioMerieux) and Mycoplasma testing using a MycoTool PCR test. Final samples taken on day 12 were additionally tested by Gram stain and Endotoxin (LAL) testing. Cell counts were obtained by conventional Trypan Blue stain and CAR-T cell content was assessed by staining cells with FITC-conjugated CD19 protein (Acro) followed by analysis on a CytoFlex cytometer (Beckman Coulter). The final product was shipped to the clinical site as a fresh (non-cryopreserved) product using the Credo cube at ambient temperature (15-25°C).

#### CAR-T cell administration

Participants received lymphodepletion with fludarabine (40 mg/m^2^ by IV daily × 3 days on days -4, -3, and -2) and cyclophosphamide (500 mg/m^2^ by IV daily × 2 days on day -4 and -3) prior to CLIC-1901 infusion. Patients were treated with a single intravenous infusion of autologous CLIC-1901 cells on day 0 at a minimum dose of 1 × 10^6^ CAR positive CLIC-1901 cells per kilogram of body weight (to a maximum of 2 × 10^8^ total CLIC-1901 cells). In participants with high disease burden, the minimum dose was reduced to 1 x 10^5^ cells/kg (with the respective maximum dose reduced to 2 × 10^7^ total CLIC-1901 cells) to reduce the risk of toxicity. High disease burden was defined as more than 20% bone marrow blasts in ALL; or a mediastinal mass greater than 1/3 of the intra-thoracic diameter on PA chest x-ray or any mass ≥ 10 cm on CT or PET scan at time of enrolment in NHL.

#### Safety and statistical analysis

An independent data safety monitoring board (DSMB) consisted of two physicians with expertise in CAR-T cells and stem cell transplantation, and one statistician. This independent DSMB met after the fourth participant received CLIC-1901 cells and had completed 28 days of follow-up, and every 6 months thereafter to review safety data including unexpected adverse events (AEs) and any deaths. Demographics and baseline characteristics are summarized for all participants. Proportions and 95% confidence intervals are summarized for dichotomous data and means, and standard deviations or medians and interquartile ranges are provided as appropriate for continuous data. Progression-free and overall survival were calculated using the Kaplan-Meier method.

### Outcomes

The primary endpoint of the study was the proportion of participants experiencing either grade 3 or 4 cytokine release syndrome (CRS), grade 3 or 4 neurotoxicity, other grade 3 or 4 toxicity (by CTCAE 4.03) or non-relapse related death within the first 28 days after CAR-T infusion. Safety outcomes included the proportion of CRS and neurotoxicity at 28 days from CLIC-1901 infusion. Grading and management of CRS and neurotoxicity was suggested by guidelines available at the time of protocol development (21). Overall response was defined as the sum of complete and partial responses. In ALL, bone marrow biopsies were performed to determine disease response and was defined by NCCN guidelines version 1.2017.42 (22). In NHL, disease assessments were made by PET-CT scan using Lugano criteria (23).

### Correlative analysis

Leukapheresis product, CAR-T cell product, and peripheral blood samples were collected, processed and biobanked for correlative analysis, with informed consent under the CLIC-01 clinical trial protocol as approved by local Research Ethics Boards. Samples were assessed for immunophenotype using multi-parameter spectral flow cytometry, for cytokine levels using the Mesoscale platform, and for CAR transgene levels using quantitative real-time PCR analysis. Please see **Supplemental Material** (S2) for detailed biobanking and correlative analysis methods.

## Results

### Patient characteristics

**Figure 1:**
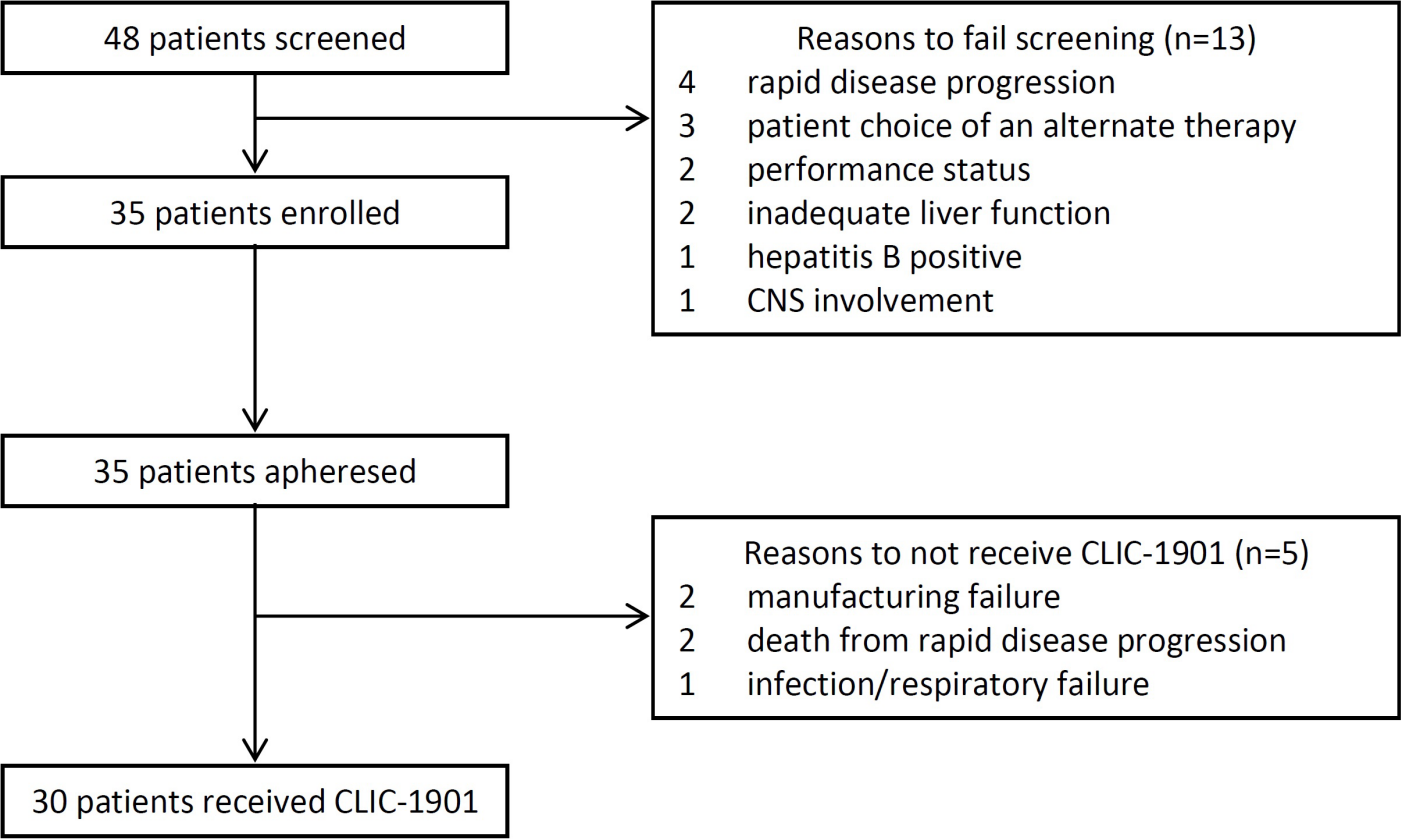
Patient Flow Diagram.

From October 1, 2019, until July 1, 2021, 48 consecutive patients were screened for eligibility, with 35 (73%) enrolled onto the study (**Figure 1**). Out of 35 participants who underwent cell collection, 30 received lymphodepleting chemotherapy and CLIC-1901 cells. Reasons for not receiving chemotherapy and CLIC-1901 cells included manufacturing failure (n=2), rapid disease progression leading to death within 2 weeks of leukapheresis (myocarditis and multiorgan failure from ALL; airway obstruction from MCL; n=2), and infection/respiratory failure in DLBCL (n=1). Characteristics of the 30 participants who received CLIC-1901 are summarized in **Table 1**. The majority were male (n=21; 70%), with a median age of 66 (range 18-75). The median number of prior therapies was 3 (range 2-6), including 13 (43%) patients who had relapsed after hematopoietic stem cell transplant (allogeneic (n=5), autologous (n=6), both (n=2)). Most participants had lymphoma (n=25) (DLBCL (n=10), MCL (n=8), DLBCL transformed from indolent lymphoma (n=4), Richter’s transformation (n=1), follicular lymphoma (n=1), plasmablastic lymphoma (n=1)), and 5 participants had B-ALL. Details regarding disease risk of the 30 participants who underwent CLIC-1901 infusion are presented in **Supplemental Table 1**. Eighteen participants had disease that was primary refractory to front-line therapy, while 2 had relapsed disease within 12 months of front-line therapy, and the remaining 8 had disease that relapsed more than 12 months after front-line therapy.

**Table 1:** Patient Characteristics for N=30 Infused.

Characteristic	N (%)
**Age (median, range)**	66 (range 18-75)
**Male sex**	21 (70)
**BMI (median, range)**	25.8 (19.7-37.3)
**Race**	
Caucasian	24 (80)
Asian	5 (16.7)
Did not disclose	1 (3.3)
**ECOG**	
0	8 (26.7)
1	19 (63.3)
2	3 (10)
**Disease**	
DLBCL	10 (33.3)
Transformed DLBCL	4 (13.3)
MCL	8 (26.7)
ALL	5 (16.7)
Other	3 (10)
**LDH at enrollment (median, range)**	245 (134-1145)
**Disease Stage (excludes ALL)**	
I	2 (6.7)
II	3 (10)
III	8 (26.7)
IV	12 (40)
**Number of prior therapies**	3 (range 2-6)
**Response to front-line therapy**	
Primary refractory	18 (60)
Relapse within 12 months	2 (6.7)
Relapse after 12 months	10 (33.3)
**Prior transplant**	
Allogeneic	5 (16.7)
Autologous	6 (20)
Both	2 (6.7)

### CAR-T cell product characteristics

The default parameters of the 12-day T cell transduction (TcT) enhanced feeding protocol that are pre-installed on the CliniMACS Prodigy by the manufacturer (Miltenyi) were used throughout, with the exception of one modification. In brief, during the pre-clinical development phase, we noted that when using the default CD4/CD8 enrichment protocol, the number of CD4/CD8 T cells that is obtained during enrichment is typically in vast excess of the required number. Thus, we calculated the volume of apheresis product containing 0.9 × 10^9^ T cells and loaded the Prodigy with this volume rather than the full volume as recommended (the Prodigy can accommodate 50-280 mL). This approach allowed the CD4/CD8 cell enrichment phase to be reduced by 1 to 3 hours (only one magnetic column pass was required instead of up to 3). All runs yielded an enriched CD4/CD8 population of sufficient quantity for subsequent steps (range of 1.64 - 11 × 10^8^ enriched CD4/CD8 cells). **Table 2** provides a summary of starting apheresis products, yield of CD4/CD8 T cells obtained from bulk apheresis during magnetic enrichment, the number of CD4/CD8 T cells used to initiate culture, and the final (day 12) product yields.

**Table 2:** Apheresis material and CAR-T cell product characteristics.

ID	Total apher.volume	# of MNC in apher.(x10^9^)	% CD3 in apher.	Vol. of apher. loaded onto Prodigy	CD4/8yield(x10^6^)	# of CD4/8 used to seed culture(x10^6^)	Total # of cells harvested on day 12(x10^8^)	% CAR positive on day 12	Total # of CAR T+ cells harvested on day 12(x10^8^)	Total # of CAR T+ cells infused(x10^8^)	Dose of CAR T cells infused(x10^6^/kg)
S001	348	10.9	13.1	219.3	660	49.5	47	36.9	17	2	3.5
S002	274	20.2	20.3	60.1	616	46.2	39	30.9	12	NI	NI
S003	291	20.0	60.3	21.7	500	50	–	–	–	MF	MF
S004	294	17.1	51.2	30.2	808	40.4	12	35.8	04	2	2.3
S005*	329	5.1	11.2	280	400	50	0.22	38.5	0.09	0.077	0.11
S006	213	7.8	34.8	70.5	716	53.7	0.38	65.3	0.25	MF	MF
S007	234	11.2	32.5	57.9	1100	113	55	28.9	16	2	2.8
S008	280	18.9	47.8	27.9	852	106.5	17	36.6	6	2	2.6
S009	288	23.9	50.9	21.3	652	97.8	35	28.3	10	2	2.7
S010	252	19	44	27.1	692	103.8	53	21.1	11	2	2.3
S011*	263	22.2	50.3	21.2	612	107.1	37	39.2	14	0.2	0.33
S012	314	17.8	15.9	99.9	564	98.7	53	33.3	18	2	2.5
S013	325	8.1	49	74.1	788	98.5	17	42.7	7	2	2.3
S014	306	29.2	56.1	16.8	536	107.2	52	29.4	15	2	2.4
S015	322	27	71.4	15.0	584	102.2	36	26.1	9	2	3.3
S016	341	13.3	81.4	28.3	820	102.5	41	20.0	8	NI	NI
S017	344	12.3	46.9	53.7	624	93.6	58	21.3	12	2	2.8
S018	278	160.9	10.1	15.4	620	93	58	21.9	13	2	2.2
S019	280	17.6	42.7	33.5	720	108	27	30.1	8	2	2.4
S020	219	9.1	43	50.6	648	97.2	49	20.2	10	2	3.6
S021*	307	20.1	33.1	41.6	628	94.2	25	18.6	5	0.2	0.32
S022	305	5.8	9.2	280.0	372	102.3	19	30.8	6	2	1.9
S023	280	34.4	3.3	220.7	624	93.6	53	24.9	13	NI	NI
S024	318	41.6	52	13.2	616	92.4	17	39.0	7	2	3.5
S025	295	19.6	66	20.5	526	105	41	16.2	7	2	2.1
S026	280	26.2	30.7	31.3	624	93.6	19	11.0	2	1.85	2.8
S027*	328	70.7	44.1	9.5	164	98.4	32	30.0	10	0.2	0.2
S028	280	30	59.4	14.1	508	101.6	33	23.2	8	2	1.7
S029	269	22	66.9	16.5	656	98.4	26	25.0	7	2	3.0
S030	280	23.7	57.1	18.6	696	104	55	32.4	18	2	2.9
S031	279	34.6	49.1	14.8	652	97.8	41	21.2	9	2	2.2
S032*	287	9.9	71.7	36.4	592	103.6	56	22.5	13	0.2	0.35
S033	280	92.5	8.5	32.1	400	100	49	23.3	11	2	1.7
S034	280	9.1	36.9	75.2	544	95.2	48	32.0	15	2	2.1
S035	330	46.5	59.8	10.7	756	94.5	55.4	26.4	15	2	2.6

*Targeted infusion dose reduced 10-fold due to high disease burden (in accordance with clinical protocol). Apher, apheresis.

The 5 gray rows represent participants not infused with CAR-T either for clinical reasons (NI) or for manufacturing failure (MF).

**Figure 2:**
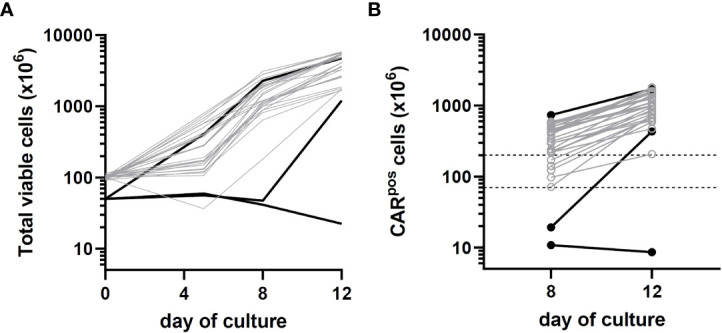
Growth kinetics of patient CLIC-1901 CAR-T cell products (n=30) manufactured using the CliniMACS Prodigy platform. CD4/CD8 T cells were enriched from bulk patient apheresis and either 50 million (black lines) or 100 million (grey lines) cells were used to initiate a 12-day culture using serum-free TexMACS medium and the ‘enhanced feeding protocol’ version of the TcT process. Cells were activated with TransAct immediately upon seeding and were transduced with lentivirus the following day. **(A)** shows the total number of viable cells present (measured on days 0, 5, 8 and 12) and **(B)** shows the number of CAR T-expressing cells present (measured on days 8 and 12). Dotted lines on **(B)** indicate the upper (200 × 10^6^) and lower (1 × 10^6^/kg; average 70 kg) numbers of CAR-T cells required for standard dosage according to clinical protocol (10-fold lower doses are required for patients with high disease burden).

**Figure 3:**
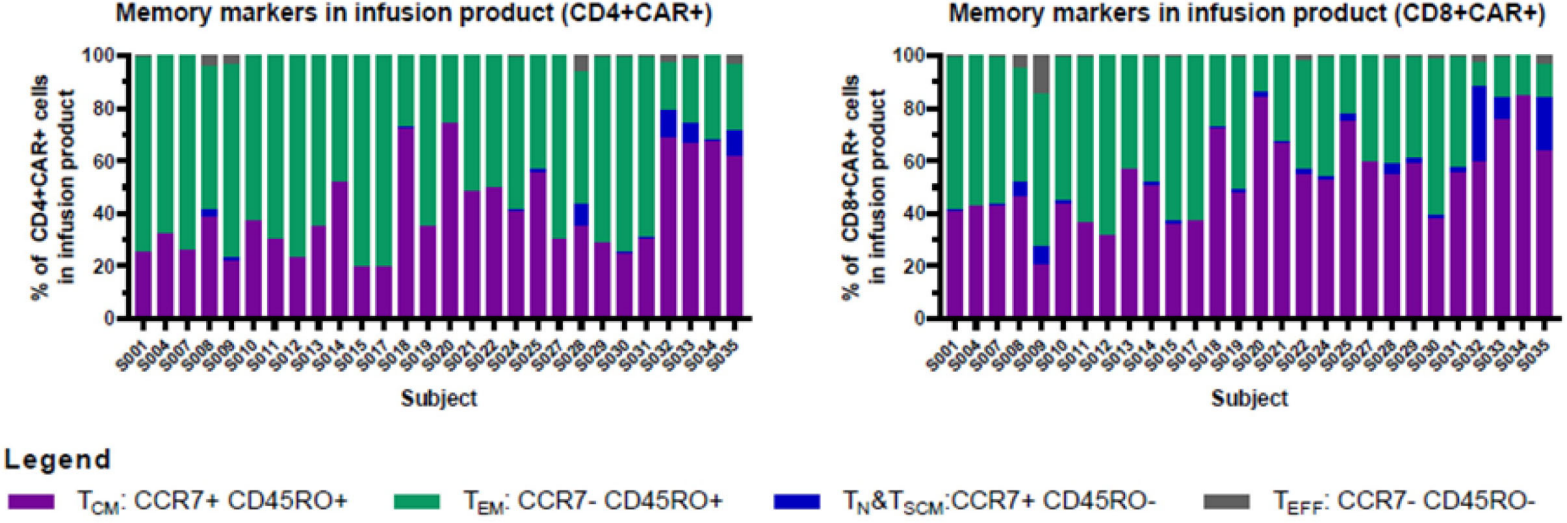
Memory markers in infusion product. CD4+ and CD8+ T cells in infusion products consisted mainly of cells with central memory or effector memory phenotypes. Cryopreserved cells were thawed, stained, and analyzed using an Aurora spectral cytometer as described in Materials and Methods. Graphs show the percentage of central memory (CCR7+ CD45RO+), effector memory (CCR7- CD45RO+), naïve/stem cell memory (CCR7+ CD45RO-), and effector (CCR7- CD45RO-) cells within the CAR+CD4+ (left), and CAR+CD8+ (right) T cell populations (gated on CD3+ CD45+ single viable cells).

For the first 6 participants enrolled on the study, culture was initiated using 5 × 10^7^ CD4/CD8 T cells, a starting number that was based upon the results of 14 pre-clinical development runs using healthy donor apheresis material. However, when this pre-clinical protocol was applied to patient apheresis materials in the trial, 3 of the first 6 runs (50%) had poor expansion, with 2 runs failing to yield sufficient numbers of CAR-T cells for infusion and one run barely meeting the minimum cell dose for high disease burden after 12 days of culture (1.1 × 10^5^ CAR-T cells/kg). We adjusted the protocol to start with 1 × 10^8^ enriched T cells, after which the manufacturing success rate was 100% (n=29), with all products demonstrating excellent *ex vivo* expansion characteristics (**Figure 2** left). In addition, one of these failures occurred when the culture was initiated with cells that had been shipped to the manufacturing site at ambient temperature and exhibited low viability in comparison to other starting cells. This prompted a change in the apheresis shipping temperature to cold shipping (1-10°C). These failures, and the subsequent 100% success rate after adjusting these parameters, suggested that seeding density and apheresis shipping temperature may be critical variables in this process. In addition, clinical factors may play a role, although our analysis did not uncover any clear clinical explanations for the manufacturing failures. The mean transduction frequency (% of cells expressing the CD19 CAR) in the final products was 28.9% (range 11 - 65.3%). Interestingly, the product with a transduction frequency of 65.3% represented one of the early manufacturing failures, suggesting that excess virus multiplicity of infection (MOI) may have been a contributing factor when starting with only 5 × 10^7^ enriched T cells. All 30 treated patients received their target dose of CLIC-1901, which was defined as a minimum of 1 × 10^6^ CAR-T cells/kg (up to a maximum of 2 × 10^8^ total CAR-T cells) for standard dose or a minimum of 1 × 10^5^ CAR-T cells/kg (up to a maximum of 2 × 10^7^ CAR-T cells) for patients with high disease burden. The actual yield of CAR-T cells on day 12 was typically in vast excess (**Figure 2** right); consequently, the majority of patients (n=28, 93%) were infused with the maximum target dose. The ratio of CD4:CD8 T cells in the final infusion product spanned a broad range (1:10 to 7:1). When comparing CD4:CD8 ratios of cell subsets within each product, the CAR+ fraction consistently contained a higher frequency of CD4+ T cells (47%) compared to CD4+ frequency in the bulk CD3+ population (36%) (paired t-test, p < 0.0001). T cell phenotypes for the infusion products are shown in **Figure 3**. The predominant memory cell phenotype was either central memory (CCR7+ CD45RO+) or effector memory (CCR7- CD45RO+) in each product.

### Clinical outcomes

#### Feasibility

Recruitment opened on Oct 1, 2019, in Ottawa and on Feb 1, 2020, in Vancouver. The first participant was screened for the trial on October 26, 2019. In the first and second year of the trial, 9 and 21 participants were infused with CLIC-1901 respectively. The beginning of the COVID-19 pandemic led to a 3-month pause in recruitment from March to May 2020. The median time from screening to enrolment was 17 days (range 0-98) which accounted for the time from signing consent to obtaining all needed testing for eligibility (bloodwork, PET scan or bone marrow biopsy, echocardiogram, and pulmonary function tests). The median time from screening to CLIC CAR-T infusion was 38 days (range 16-117). The median time from enrolment to apheresis was 4 days (range 0-33) and enrolment to CAR-T infusion was 19 days (range 15-48 days). The median time from leukapheresis to CAR-T infusion was 15 days (range 13-16 days for fresh product, one participant received frozen product at 28 days from leukapheresis). The median time from last disease progression to eligibility was 47 days (range 10-183), and was notably longer for MCL (median 88 days, range 26-183 days). Otherwise, there were no differences in median times described by disease group. In summary, accrual and time from progression to enrollment and CAR-T infusion was deemed feasible and appropriate for this patient population.

#### Safety

A total of 7 out of 30 participants (23.3%) had grade 3 or 4 CRS, grade 3 or 4 neurotoxicity, other grade 3 or 4 toxicity or non-relapse related death within the first 28 days after CAR-T infusion (the defined primary endpoint). CRS was observed in 18 participants (60%) at a median onset of 1.5 days after CLIC-1901 infusion (range 0-9 days). Two participants (7%) experienced grade ≥ 3 CRS; of note, these were the only 2 participants on the study to have any grade of neurotoxicity. One participant with primary refractory DLBCL developed CRS grade 3 on day one, and subsequently developed concomitant grade 2 neurotoxicity on day 4, both of which resolved on day 7. This participant had significant bowel involvement of lymphoma and ultimately died on day 28 from a bowel perforation. The other participant had B-ALL and developed grade 1 CRS on day 0 which progressed to grade 3 by day 5 with onset of grade 4 neurotoxicity on day 5 as well. The patient ultimately died on day 9 from CRS and multiorgan failure, without resolution of neurotoxicity. Adverse events occurring in more than one participant per CTCAE version 4.03 are reported in **Table 3**. Two patients died from non-relapse causes within the first 28 days of receiving CLIC-1901 CAR-T cells. These are the same 2 participants who had high-grade CRS and neurotoxicity as described above.

**Table 3:** Adverse Event Data to 30 days after CLIC-1901 Infusion.

Adverse Event	Any grade(N patients, %)	Grade ≥ 3(N patients, %)
**CRS**	18 (60%)	2 (6.7%)
**Neurotoxicity**	2 (6.7%)	1 (3.3%)
Vomiting	11	0
Edema	10	2
Anorexia	8	0
Nausea	8	0
Diarrhea	7	0
Headache	6	0
Abdominal pain	5	1
Fatigue	5	0
Febrile Neutropenia	5	4
Pain	5	0
Rash maculo-papular	5	0
Tachycardia	5	0
Bruising	4	0
Constipation	4	0
Decreased fibrinogen	4	0
Dizziness	4	0
Infection	4	0
Chills	3	0
Hypokalemia	3	0
Hypotension (*not CRS)	3	0
Rigors	3	0
Tremors	3	0
Cough	2	0
Dehydration	2	0
Dyspepsia	2	0
Dyspnea	2	0
Increased INR	2	1
Memory impairment	2	0
Pruritus	2	0
Urticaria	2	0
Weight Gain	2	0

Persistent cytopenias are a well-known side effect after CD19 CAR-T cell therapy (2, 24). Cytopenias were graded using CTCAE version 4.03. In brief, of the 28 participants who were evaluable at day 28, 2 (7%) had grade 3 anemia, 10 (36%) had severe neutropenia (n=5; grade 3, n=5; grade 4) and 10 (36%) had severe thrombocytopenia (n=2; grade 3, n=8; grade 4). By month 3, of the 20 evaluable patients, zero participants had severe anemia, 5 (25%) participants had severe neutropenia (all grade 3) and 3 (15%) had severe thrombocytopenia (all grade 4). At month 6, of 13 evaluable patients, 2 (15%) had grade 3 neutropenia, 1 (8%) had grade 3 thrombocytopenia and 1 (8%) had grade 4 thrombocytopenia. The participants with ongoing severe neutropenia were not the same as the participants who had severe thrombocytopenia. Two participants had bone marrow biopsies to assess for ongoing cytopenias (one with marrow-based lymphoma and one with B-ALL), one case showed a hypocellular marrow and the other showed a hypercellular marrow, but no evidence of disease or myelodysplasia in either biopsy.

#### Efficacy

**Figure 4:**
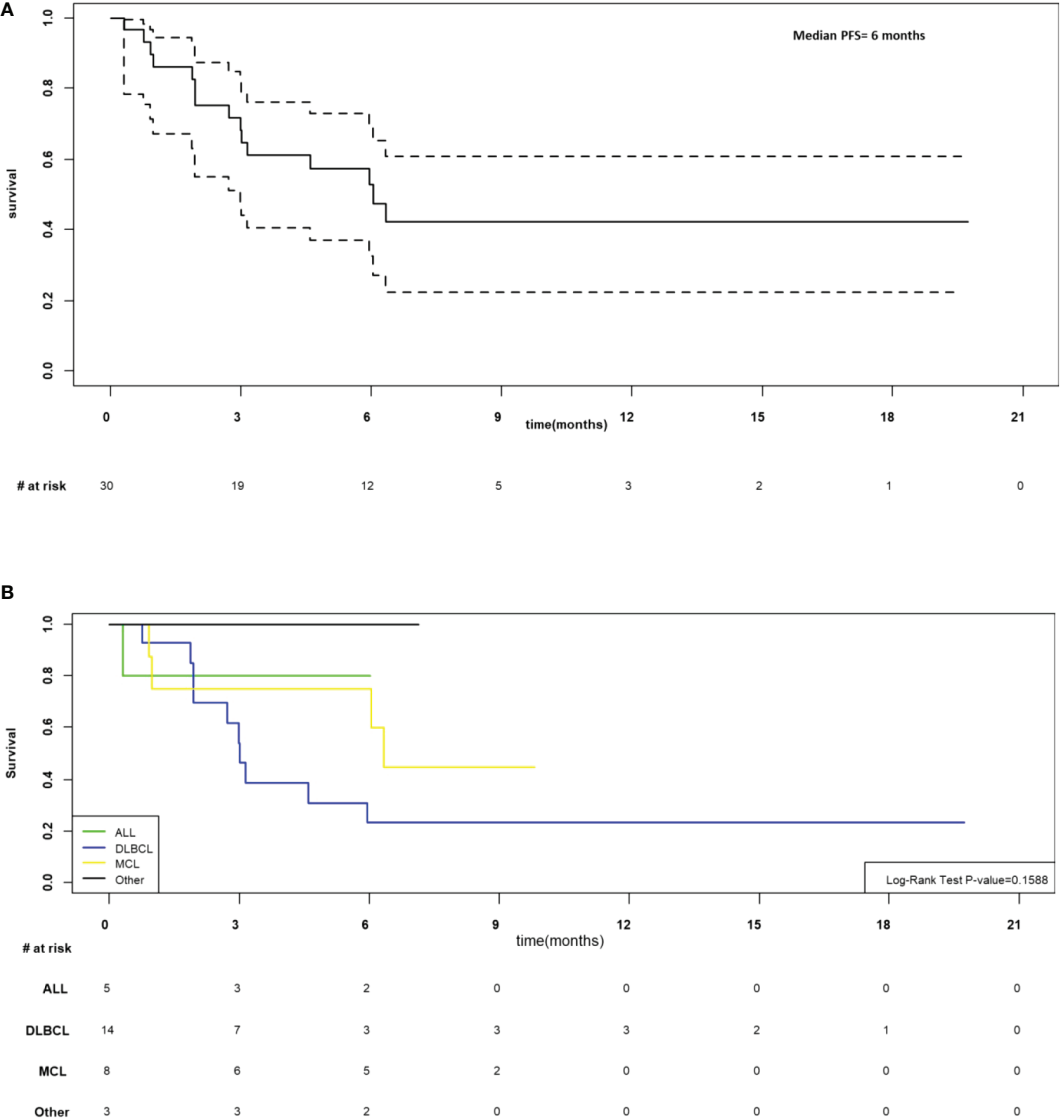
**(A)** Progression-free survival for N=30 infused. **(B)** Progression free survival by disease.

**Figure 5:**
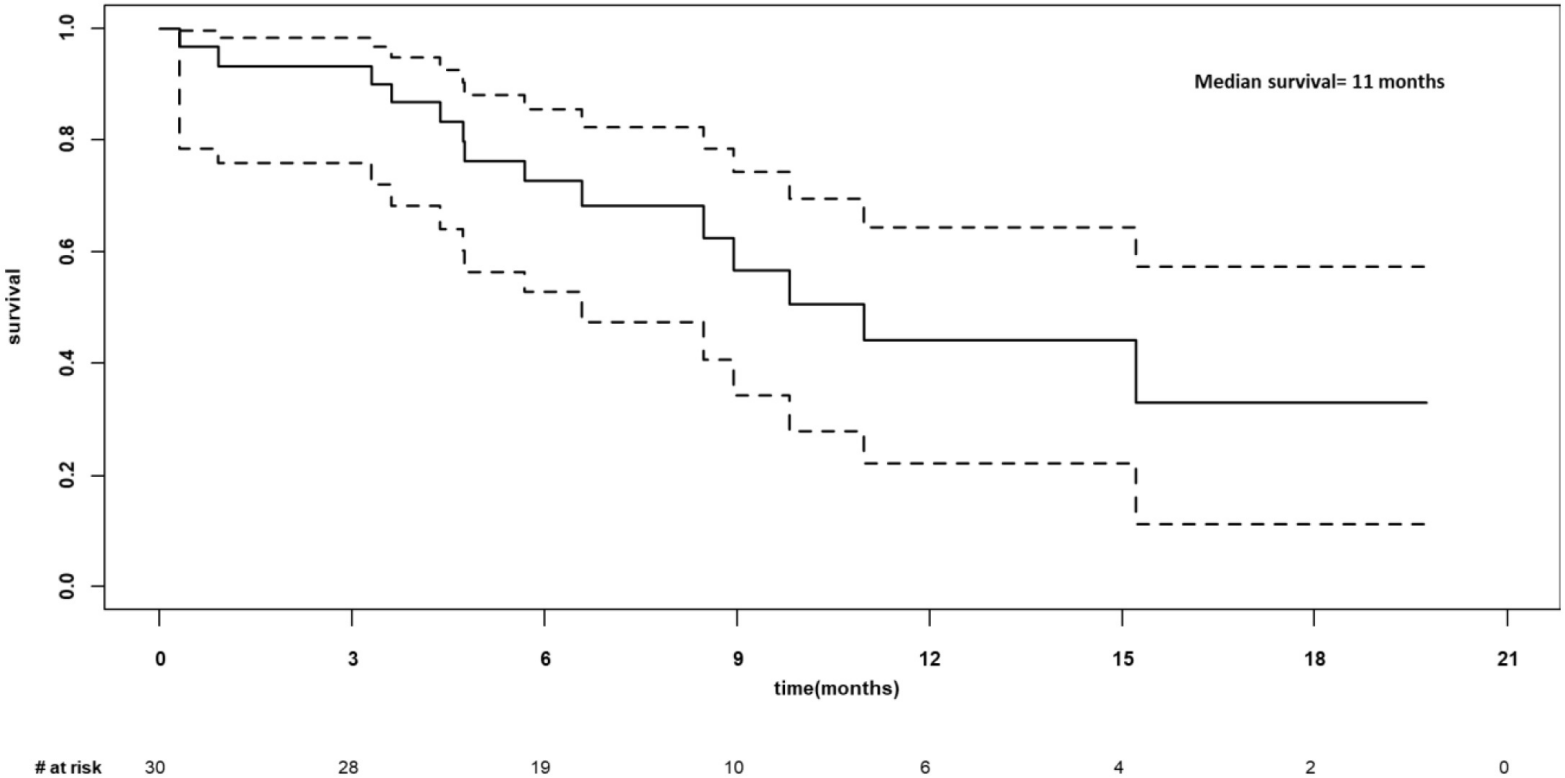
Overall survival for N=30 infused.

**Figure 6:**
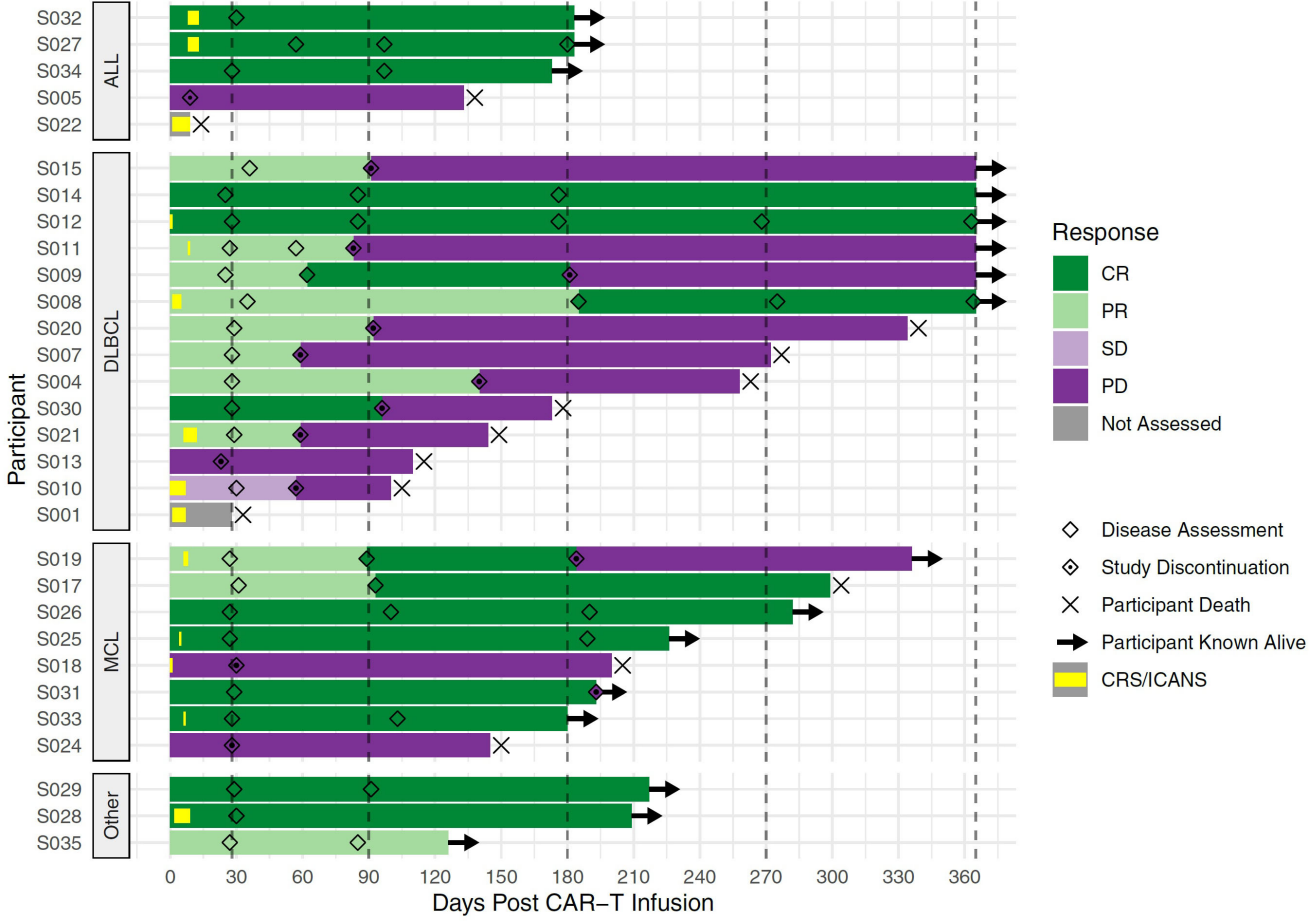
Swimmer’s Plot of Patient-level Data. Each bar represents one CLIC-01 participant infused with CLIC-1901. Participants are grouped into disease type (ALL, DLBCL, MCL, and Other). X-axis denotes the days after CAR-T infusion. Vertical dashed lines mark expected disease assessment time points. Hollow diamonds indicate the days where a disease assessment was made for each participant. Bars are coloured according to the response at each disease assessment, with the time between 2 assessments being coloured by the earlier assessment time. For the time before the first assessment, bars are coloured by the response observed at the first assessment. Periods of CRS or neurotoxicity are denoted by yellow bars within the overall bar. An × marks the death of a participant, and → denotes the patient is still known to be alive at that time point. For this figure, follow up is truncated at 1 year.

As of March 7, 2022, the median follow-up for the 30 participants was 6.5 months (205 days; range 9-601 days). In the 28 participants evaluable at day 28, the overall response rate was 76.7% (CR n=12, PR n=11, SD n=1 and PD n=4). The median progression-free was 6 months (95%CI 3-not estimable, NE; **Figure 4A**) and appeared to be best in ALL (**Figure 4B**). The median overall survival was 11 months (95% CI 6.6-NE), **Figure 5**. The median PFS and OS for DLBCL was 3 months (95%CI 1.9-5.9) and 10 months (95%CI 3.6-NE) respectively. The median PFS and OS for MCL was 6.3 months (95%CI 0.9-NE) and 9.8 months (95%CI 4.8-NE) respectively. The median PFS and OS was not estimable for ALL or other diagnosis. Patient level data is presented in **Figure 6**.

### Correlative analysis

**Figure 7:**
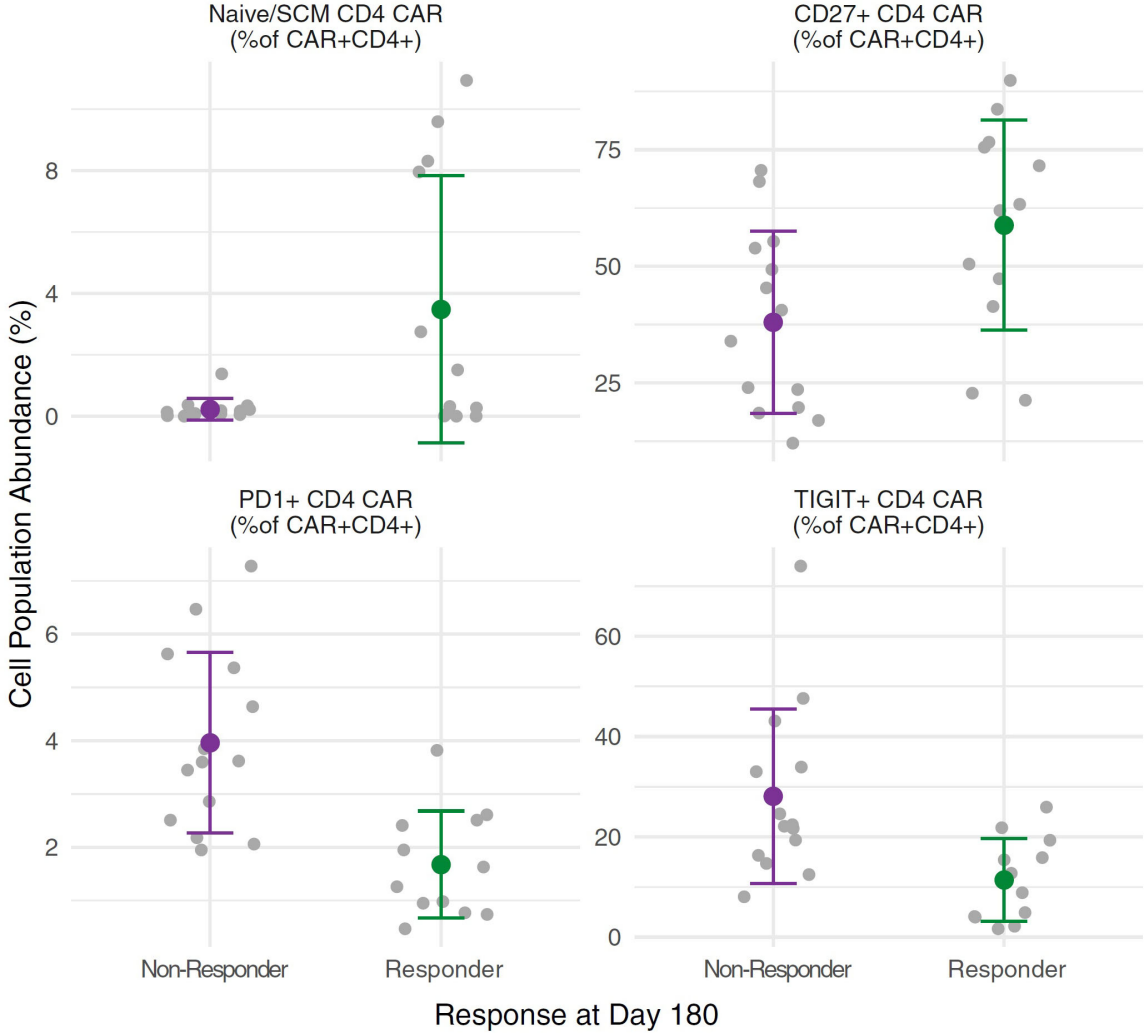
CAR cell phenotypes in the infusion product for responders versus non-responders. Independent ANOVAs were run to test for differences in CAR-T cell phenotypes between responders (n = 12) and non-responders (n = 14) at day 180, with Benjamini & Hochberg (BH) multiple test correction. Increased frequencies of Naïve/SCM CD4+ CAR-T cells (ANOVA p = 0.010, BH-adjusted p = 0.077) and CD27+ CD4+ CAR-T cells (ANOVA p = 0.019, BH-adjusted p = 0.107) in the infusion product correlated with favourable responses at day 180, as did decreased frequencies of PD1+ CD4+ CAR-T cells (ANOVA p = 0.00041, BH-adjusted p = 0.009) and TIGIT+ CD4+ CAR-T cells (ANOVA p = 0.0057, BH-adjusted p = 0.066). For each cell subset, grey points show individual measurements for each subject, while the green (responders) or purple (non-responders) points show the mean with whiskers extending to the mean ± standard deviation for each group.

We performed multi-parameter flow cytometry analysis of patient PBMC isolated from peripheral blood samples drawn at the time of apheresis, and of the infusion products. There was a nominally significant association between CD28 expression on CD4+ T cells and response (CR or PR) at day 180 after CAR-T cell infusion (91.3% CD28+, range 56.9–99.9% for responders versus 79.4%, range 44.6–99.9% for non-responders, p=0.048, t-test), but no other statistically significant associations between clinical outcomes and the frequencies of immune cell subsets in PBMC samples or the infusion products. We then compared the total number of cells infused to overall response at day 180. This was of interest because dose is defined by the number of CAR positive T cells such that products with lower CAR-T content have greater numbers of total infused cells; however, no significant correlation was observed (p = 0.304, t-test). We then compared the phenotypes of CAR-T cells in the infusion product to overall response and noted that favourable response at day 180 was associated with increased frequency of naïve/SCM CD4+ CAR-T cells (ANOVA p = 0.010, BH-adjusted p = 0.077), increased frequency of CD27+ CD4+ CAR-T cells (ANOVA p = 0.019, BH-adjusted p = 0.107), decreased frequencies of PD1+ CD4+ CAR-T cells (ANOVA p = 0.00041, BH-adjusted p = 0.009) and decreased frequencies of TIGIT+ CD4+ CAR-T cells (ANOVA p = 0.0057, BH-adjusted p = 0.066) in the infusion product (**Figure 7**). These findings are consistent with previous reports of CAR-T cell phenotypes associated with favourable outcomes.

We then evaluated B cell persistence and CAR-T persistence in the blood at days 3, 7, 14, 28, month 2 and month 3. As measured by flow cytometry, we saw the level of CAR-T cells in the blood peak at day 7 or 14 and typically saw B cell levels drop at day 14 (**Supplemental Figure 1**). To give an independent and sensitive measure of CAR-T abundance in participant blood over time, and to provide data for time points where there were not enough cells to analyze by flow cytometry, we developed a qPCR assay to quantify the CAR-T transgene in total nucleic acid extracted from PBMC. **Supplemental Figure 2** shows the results from this assay for the first 90 days, which was largely consistent with CAR-T cell frequencies obtained by flow cytometry. We did not observe any correlations between CAR-T cell expansion dynamics and participant outcomes. Finally, we measured cytokine levels in patient serum during the first 28 days. In general, we saw trends towards increased cytokine levels at days 3 and 7 compared to pre-infusion time points, and in patients that experienced CRS grade 3 or greater. The largest increases were seen with GM-CSF, IFN-g, and IL-6, and to a lesser extent, IL-1b and TNF-b (**Supplemental Figure 3**). However, these trends in cytokine levels did not reach statistical significance. While the correlative data presented here are consistent with favourable immune activation in study participants, there is insufficient power to link specific markers to outcomes. We will conduct a more extensive correlative analysis when accrual to the study is complete.

## Discussion

Here we report our first experience with an in-house manufactured non-cryopreserved CAR-T cell product. Consistent with previous studies, we demonstrate that CAR-T cell manufacturing using the Miltenyi Prodigy platform is feasible in an academic setting. In this trial, we had two early manufacturing failures, both of which were due to a process design issue that was successfully addressed leading to zero further failures. We were able to provide fresh (i.e. non-cryopreserved) CAR-T product, with a 12-day manufacturing platform and a rapid 15-day turnaround from apheresis to CAR-T infusion. This is significantly shorter than the 4-to-6-week manufacturing and turnaround of commercial CAR-T products that we experience at our centers, and significantly shorter than other reported in-house manufacturing efforts (25). Our short manufacturing time allowed all patients to avoid the need for bridging therapy between apheresis and CAR-T infusion, which has been associated with poor clinical outcomes with CAR-T cell treatment (26).

Our trial showed that despite the COVID-19 pandemic and large geographical size of Canada, non-cryopreserved cells could be transported by volunteer couriers to and from our manufacturing facility (Victoria, British Columbia) and two clinical sites over 4,300 km apart (Vancouver, British Columbia and Ottawa, Ontario). Thus, we successfully leveraged the current standard of care for transportation of hematopoietic stem cell transplant products for Canada to achieve shorter vein-to-vein time than previous studies.

Lastly, in this early analysis of 30 patients treated on trial, safety and efficacy results were comparable to early phase trials performed for the other CD19 CAR-T products. In the ZUMA-1 trial of 111 patients with DLBCL who were treated with axicabtagene ciloleucel, grade 3 or higher CRS or neurotoxicity was 13% and 28% respectively (2). In the JULIET trial which reported the results of 167 patients and led to the approval of tisagenlecleucel for clinical use in DLBCL, the rate of grade 3 or higher CRS was 23% and the rate of severe neurotoxicity was 11% (3). In this CLIC-01 study, only 2 of the first 30 patients infused experienced grade 3 or higher CRS and neurotoxicity, giving this trial a low rate of 7% for these common CAR-T cell toxicities. We did not find a correlation between patient characteristics or infusion product characteristics in predicting toxicity, but with such a low incidence of CRS and neurotoxicity in this first group of patients, we are likely underpowered to report this at present. Our reported median progression-free survival is 6 months, which cannot at this time be compared to commercial products as this includes multiple disease types. In DLBCL, the median PFS in this trial was 3 months which does not differ significantly from the JULIET (2.9 months) and ZUMA-1 (5.9 months) trials (2, 3). Our experience in this trial is difficult to compare directly to real-world data with commercial products as we found that patients were often referred to the CLIC-01 trial because they were considered too sick (due to rapid disease progression or performance status) to wait for commercial CAR-T funding approvals and manufacturing times.

While this study provides promise for an in-house manufactured CD19 CAR-T product, there are limitations. With only 30 patients reported here and short follow-up, we cannot draw firm conclusions about safety and efficacy. In addition, the trial is currently underpowered to provide conclusive correlative results to better predict for toxicity, CAR-T persistence, and overall response. With multiple diseases included in this analysis, it is also not possible to conclude what the response rate is for each specific disease type. Nonetheless, this trial demonstrates that non-commercial organizations, working together, can effectively and efficiently manufacture in-house, high-quality CAR-T products for distribution and administration across a network of clinical sites. While this is a large undertaking for academic institutions, as has been outlined elsewhere (27), it also represents an opportunity to reduce costs and improve efficiency of manufacturing processes for future CAR-T therapies. While we must estimate the cost of our innovative platform and measure how this may change over time, there is compelling evidence (28–30) that our platform could fix the cost-prohibitive nature of commercial products that have placed immense pressure on publicly funded health care systems like Canada. In addition, our platform will not only provide a feasible approach with fast manufacturing for a conventional CAR-T, but it will also allow for flexibility of building the next CAR-T product, due to the rapid vector and CAR development which is not restricted by meeting world-wide supply and demand constraints of pharmaceutical companies, allowing for more rapid improvement in patient outcomes with CAR-T therapies.

## Data availability statement

The raw data supporting the conclusions of this article will be made available by the authors, without undue reservation.

## Ethics statement

The studies involving human participants were reviewed and approved by Ottawa Health Science Network Research Ethics Board (OHSN-REB) and The University of British Columbia - BC Cancer Research Ethics Board. The patients/participants provided their written informed consent to participate in this study.

## Author contributions

NK, RAH, HA, JB, BN, ML, DF, JP, and KT designed the research. RM and SB analyzed data. JW, JN, EY, LD, NG, KC, TD, RH, BL, RF, VH, HL, LMC, JQ, PY, and DV were responsible for process development and manufacturing of the products. MB, LM, DC, MG, SB, CM, AS and EW provided quality and regulatory oversight. NK, KH, A-MC, AB, SM, MK, LH, KS, SN and JR conducted the clinical trial. JN, LD, DW and JS designed and executed immune monitoring assays. MS, DB and SY provided project management and administrative support. SD and TH provided patient perspective for the study design and consent. All authors contributed to the article and approved the submitted version.
