# Supplementary material for: CLIC-01: Manufacture and distribution of non-cryopreserved CAR-T cells for patients with CD19 positive hematologic malignancies
Source: Front Immunol. 2022 Dec 19;13:1074740. doi: 10.3389/fimmu.2022.1074740 (PMC9806210; doi:10.3389/fimmu.2022.1074740)
Supplement: Supplementary file 1 [file DataSheet_1.pdf]

Supplemental Material

S1. Detailed manufacturing methods

Lentivector Manufacturing. 293T/17 cells were passaged in Dulbecco's Modified Eagle Medium (DMEM; Invitrogen) containing 10% fetal bovine serum (FBS; Invitrogen), and 25mM HEPES (Invitrogen), and expanded into 36-layer HYPERstacks (18,000 cm2 per 36-layer HYPERstack) (Corning). After three cell passages, 6 × 36-layer HYPERstacks were transfected with the three plasmids using TransIT-Lenti transfection reagent (Mirus Bio) to generate a total crude harvest volume of approximately 24 L (3.9 L of media harvested per 36-layer HYPERstack). Crude supernatant was clarified to remove cell debris, treated with benzonase to remove residual cellular DNA, and concentrated via tangential flow filtration (TFF) from ~24 L to 2.4 L. At the 2.4 L volume, diafiltration was performed to formulate the CD19 CAR lentivirus in TexMACS Medium (Miltenyi). CD19-CAR lentivirus was further concentrated using high-speed centrifugation, resuspended in TexMACS buffer (Miltenyi) and then aliquoted into 2 mL cryovials at 500 µL/vial and stored at OHRI at -60°C to -90°C. Lentiviral titer on the frozen virus product was assessed using quantitative PCR (qPCR), yielding a titer of 1.8×10^7 Transduction Units (TU)/mL with a total yield of 3.0×10^9 Total TU. The following release tests were performed on the final CD19CAR lentiviral product: 1) host cell DNA by qPCR, 2) residual benzonase quantitation, 3) residual VSV-G plasmid, E1A, or SV40, 4) endotoxin level, 5) sterility, 6) pH & appearance, 7) identity, and 8) titer assay. Additionally, the replication competent lentivirus release test was performed on the crude lentivirus harvest, and four release tests (for mycoplasma, 9 CFR Bovine Virus, adventitious viral contaminants, and replication competent lentivirus) were performed on the control cells in spent media.

***CAR-T Cell Manufacturing*.** Patients were apheresed using a standard MNC collection procedure. Immediately following apheresis collection, the product was packaged in a standard transfer device (Credo cube) and shipped without cryopreservation from the apheresis location (Ottawa, Canada or Vancouver, Canada) to the cell processing facility in Victoria, Canada. Shipping was performed using an established volunteer-based courier system wherein a designated individual personally picked up, transported and delivered the product, thereby maintaining a continuous chain of custody. Transit times were either overnight (Ottawa) or same day (Vancouver). For the first four patients on the trial, products were maintained at ambient temperature (15-25°C) during shipment. However, two of these products exhibited reduced viability, leading us to change the shipping conditions from an ambient to refrigerated temperature (1-10°C) using the frozen TIC plates of the Credo Cube cooler. Temperatures within the Credo shipping device were continuously monitored using electronic temperature monitoring devices. Upon arrival at the processing center, apheresis products were stored in the Credo shipping device until manufacturing was initiated, typically the morning after product arrival and no more than 46 hours from the completion of apheresis, as per the standard operating procedure (SOP).

CAR-T cells were manufactured using the Miltenyi CliniMACS Prodigy system installed in a classified Grade D manufacturing suite. Any 'functionally open' steps (i.e., media preparation and cell manipulation) were performed in a classified Grade A isolator (NuAire). We followed

the ‘enhanced feeding protocol’ version of the pre-installed T cell transduction (TcT) protocol, which is designed for serum-free cultivation. In brief, the process was performed over a fixed window of 12 days, following a set of semi-automated processes comprised of 1) CD4/CD8 T cell enrichment, culture initiation and T cell activation (day 0); 2) lentivirus transduction (day 1); 3) lentivirus washout (day 3); 4) feeding and on-demand in-process sampling (days 3-11); and 5) cell washing and culture harvest (day 12). The TcT process (as installed) requires a starting volume of 50-280 mL of apheresis material and, depending on the number of T cells present, performs up to three magnetic enrichment steps, using 1×10^9 T cells per step. However, as each magnetic enrichment step takes approximately one hour to complete and only 1×10^8 T cells are required to initiate culture, we modified the protocol to load only the volume of apheresis material containing 0.9×10^9 T cells, which resulted in one magnetic enrichment pass. This involved either removing excess apheresis volume from the original apheresis bag or removing the volume of material containing 0.9×10^9 T cells into a new bag and adding sterile CliniMACS wash buffer (PBS with 0.5% human serum albumin [HSA]) to the minimum required volume of 50 mL. Apheresis material was attached to the pre-installed CliniMACS TS520 tubing set via sterile welding (Terumo TSCD II). Following magnetic enrichment, CD4/CD8 T cells were counted and culture was initiated using 5×10^7 (patients #1-6) or 1×10^8 (patients #7-35) enriched T cells in serum-free TexMACS medium (Miltenyi) containing IL-7 and IL-15 (12.5 ng/mL each) along with 5 ug/mL gentamicin. When preparing culture media on day 0, 10 mL of prepared media was removed from the media bag and reserved for diluting the CAR-encoding lentivirus on day one.

On day one, lentivirus (in cryovials) was thawed, and 0.5 mL of lentivirus stock and 10 mL of media was transferred to a CS50 bag within the Grade A isolator. The bag was then sealed, removed from the isolator and attached via sterile welding to the installed CliniMACS tubing set. On day five, a second bag of serum-free TexMACS medium containing IL-7, IL-15, and gentamicin was prepared and the initial (depleted) media bag was removed, just prior to the media feed scheduled for day five. After the media feed was completed, an in-process sample was obtained and subjected to in-process sterility and mycoplasma testing, the results of which were used as release criteria for final product on day 12. On day eight, an in-process sample was obtained and assessed for CAR-T cell content by flow cytometry. If at least 1×10^7 CAR-T cells were present, notice was given to the clinical site to initiate lymphodepleting chemotherapy.

On day 12, an in-process sample was obtained and assessed for CAR-T cell content by flow cytometry. While CAR staining was underway, the remaining cell product was washed and harvested into 100 mL sterile Plasmalyte A containing 0.5% HSA). The final cell product was harvested using the target cell bag of the TS520 tubing set with an upstream in-line filter to remove any cell clumps. The target cell bag was removed from the TS520 tubing set using the integrated line sealer and sterile welded to either CS50N or CS250N bags (Origen), depending on the volume of final product. The combined target cell bag/CS50N/CS250N bag was then transferred into a grade A isolator for preparation of the final drug product.

The volume of cell suspension containing the required CAR-T cell dose was calculated (based on total cell density and percent transduction) and the required volume of cell suspension (plus an extra 10 mL for QC assessment) was aseptically transferred to the CS50N/CS250N final product bag via syringe transfer, maintaining the functionally closed status of the product bag. Ten mL of the final product was then removed for QC assessment purposes and the bag was then hermetically sealed and labeled. The final product was then shipped to the clinical site as a fresh (non-cryopreserved) product using the Credo cube and volunteer courier system, as described above, except that the product was maintained at ambient temperature (15-25°C) during the return shipment. Stability studies showed that cells could be maintained in this solution for up to 48 hours at room temperature with only a marginal decline in viability (data not shown). All products were delivered to the clinical site in a fresh (non-cryopreserved) format, with the exception of one product that was cryopreserved due to patient ineligibility on the planned day of infusion. All products passed QC review prior to release for infusion (in-process sterility, in-process mycoplasma, gram stain, endotoxin and CD3 content > 90%). In one instance, the patient was not eligible to receive infusion on the designated day for clinical reasons, so the product was cryopreserved to accommodate a different infusion date. In this instance the day 12 process was identical to that described above, except that the final product was mixed 1:1 with CryoStor CS10 (BioLife), and cells were frozen in the CS50N bag using a Planer controlled rate freezer. After being stored in the vapor phase of a nitrogen shipper, cells were shipped to the clinical site using a nitrogen dry shipper and thawed at the patient bedside prior to infusion.

In-process control samples obtained on day 5 and final product samples obtained on day 12 were subjected to sterility testing using a BacT ALERT system (BioMerieux) and Mycoplasma testing using a MycoTool PCR test. Final samples taken on day 12 were additionally tested by Gram stain and Endotoxin (LAL) testing. Cell counts were obtained by conventional Trypan Blue stain and CAR-T cell content was assessed by staining cells with FITC-conjugated CD19 protein (Acro) followed by analysis on a CytoFlex cytometer (Beckman Coulter).

S2. Correlative analysis detailed methods

Biobanking. Peripheral blood samples were collected at multiple time points with informed consent under CLIC-01 clinical trial protocols as approved by local Research Ethics Boards. Sample processing was initiated within 3 hours of collection. SST tubes were processed using standard protocols. Sodium heparin tubes were first centrifuged to obtain plasma, and then remaining material was processed over a ficoll density gradient to obtain peripheral blood mononuclear cells (PBMC). One million cells were resuspended in RLT + 4% TCEP, frozen, and shipped to Canada's Michael Smith Genome Sciences Centre for total nucleic acid extraction. Remaining cells were cryopreserved in CryoStor, and shipped, along with frozen serum and plasma, to the CLIC Biorepository at BC Cancer's Deeley Research Centre. PBMC were stored in nitrogen vapour freezers, and all other samples were stored in -80°C freezers. Sample processing and storage information were added to a secure online database (CTRNet ATiM).

Supplemental Material

Flow Cytometry. Cryopreserved PBMC and final infusion products were thawed and washed in TexMACS Medium (Miltenyi) containing gentamicin (Sandoz), and then stained with the Zombie NIR Fixable Viability Kit (BioLegend) in serum-free phosphate-buffered saline (PBS; Gibco). After washing, samples were blocked with Human TruStain FcX (BioLegend) in FACS buffer (2% FBS in PBS) and then stained in FACS buffer containing BD Horizon Brilliant Stain Buffer Plus (BD Biosciences) using the antibodies listed in Supplemental Table 2. After washing with FACS buffer, cells were stained with FITC-conjugated CD19 protein (AcroBio, catalogue # CD9-HF251) to label CAR-positive cells. Samples were run on an Aurora spectral flow cytometer (Cytek). Data were unmixed using SpectroFlo Software (Cytek) and imported to FlowJo (BD) for analysis.

Cytokine analysis. Serum cytokine measurements were performed using custom ten-analyte Mesoscale V-Plex Plus kits following the manufacturer's alternate protocol #1 (Cat#K151A9H-2, Mesoscale Discovery, Rockville, MD). All cytokine measurements were performed in duplicate on biobanked serum aliquots subjected to a single freeze-thaw and diluted two-fold into assay-specific diluent prior to measurement according to the manufacturer's recommendations. Samples were retested whenever the coefficient of variation (CV) of duplicate measurements exceeded 20%. Samples that demonstrated results above the assay range for any of the measured cytokine analytes were retested using 100- or 1000-fold dilution into assay-specific diluent. Electrochemiluminescence measurement of plates at assay completion was performed using a MESO QuickPlex SQ 120 imager and data analysis was performed using MSD Workbench Software Version 4·0 (Mesoscale Discovery, Rockville, MD).

Quantitative PCR analysis of CAR copy number. Patient PBMC were pelleted and resuspended in lysis buffer (RLT Plus Buffer (Qiagen) + TCEP (Sigma Aldrich)) for total nucleic acid (TNA; DNA and RNA) extraction using the EvoPure RNA isolation kit (ALine Biosciences) automated on a Microlab NIMBUS (Hamilton) liquid handler. The DNA component of the TNA was quantified using Qubit dsDNA BR Assay Kit (Thermofisher). DNA derived from patient PBMCs was then interrogated for *CD19CAR* transgene integration using qPCR assay targeting CD19CAR sequence as well as the normalization target *CDKN1*. Briefly, a plasmid standard appropriate for multiplexing was generated by cloning a cassette containing *CDKN1* into a plasmid containing CD19CAR. The resulting construct was maxiprepped using Purelink™ Expi Endotoxin-Free Maxi Plasmid Purification Kit (Thermofisher), and linearized using NotI-HF restriction enzyme (New England Biolabs). Digested DNA was quantified in quadruplicate using Qubit dsDNA BR Assay Kit (Thermofisher). Quantitative PCR analysis was performed on TNA samples (100 ng DNA per reaction), using 2X Taqman Fast Advanced Master Mix (Thermofisher) and run on the Quant Studio 6 Flex (Applied Biosystems). *CD19CAR* was detected using a custom Taqman primer/probe set targeting the junction between the 4-1BB and ζ signaling chains as described (Milone, M.C., et al., Chimeric receptors containing CD137 signal transduction domains mediate enhanced survival of T cells and increased antileukemic efficacy in vivo. Mol Ther, 2009. 17(8): p. 1453-64).

CD19 4-1BB F primer: 5′-TGCCGATTTCCAGAAGAAGAAGAAG-3′
CD19 4-1BB R primer: 5′-GCGCTCCTGCTGAACTTC-3′
CD19 4-1BB MGB probe: 5′-VIC-ACTCTCAGTTCACATCCTC-3′

A custom Taqman primer/probe set targeting *CDKN1* (Kalos, M., et al., T cells with chimeric antigen receptors have potent antitumor effects and can establish memory in patients with advanced leukemia. Sci Transl Med, 2011. 3(95): p. 95ra73.) was used to determine number of cells per reaction:

CDKN1 F primer: 5'-GAAAGCTGACTGCCCCTATTTG-3'
CDKN1 R primer: 5'-GAGAGGAAGTGCTGGGAACAAT-3'
CDKN1 MGB probe: 5'-FAM-CTCCCCAGTCTCTTT-3'

Final concentrations were 900 nM each F/R primers, 250 nM probe. A 5-point standard curve was generated consisting of each order of magnitude from 10^2 to 10^6 copies of control linearized plasmid spiked into 100 ng DNA of control murine cell line EL4 (TIB-39, ATCC) TNA per reaction. Each data point (samples, reference samples, standard curve) was evaluated in quadruplicate with mean values and standard deviations reported. The mean number of CAR copies per cell was calculated by dividing mean *CD19CAR* values by the number of cells (*CDKN1* mean ÷ 2).

The qPCR assay was validated using two independent *CD19CAR* positive cell banks: a Jurkat CAR control cell line, and a CAR T Prodigy cell bank, prepared as follows. Human leukemic T cell lymphoblast Jurkat cells (clone E6-1, TIB-152™, ATCC) were transduced with a *CD19CAR* lentivirus at low MOI (0.07) to reduce the chances of > 1 integration events/cell. Transduced cells went through two rounds of expansion and were then sorted for CD19CAR protein expression to obtain a positive population. Sorting was performed on the FACS Aria III (BD) using 1:25 dilution Goat anti-mouse IgG-AF647 (Jackson 115-606-072) to stain the CD19CAR protein. The purity (98.5%) of the resulting population was ascertained by running samples stained with Goat anti-mouse IgG-AF647 on the Fortessa I (BD). In addition, a primary CAR-T cell bank was prepared for the purpose of qPCR method development. This was generated from a healthy donor leukopak (Hemacare) using the CliniMacs Prodigy instrument (Miltenyi) and T cell transduction (TcT) protocol. In brief, CD4 and CD8 lymphocytes were purified from the bulk leukopak material via magnetic selection and 50 million enriched CD4/CD8 T cells were used to initiate a TcT culture in serum-free TexMacs media containing 12.5 ng/mL IL-7 and 12.5 ng/mL IL-15 (Miltenyi). On day 0 of the culture T cells were activated with one vial of CD3/CD28 TransAct reagent (Miltenyi), and on day one of the culture cells were transduced with a *CD19CAR* lentivirus construct at an MOI of 0.32. Cells were expanded for a total of 12 days and then harvested into Plasmalyte (Baxter) containing 0.5% human serum albumin (CSL Behring). Cells were cryopreserved as single use aliquots at 1×10^6 cells/mL in CryostorCS10 (BioLife) and stored in vapor phase nitrogen. The CAR T cell bank was qualified by assessing twenty randomly chosen vials for CAR expression by staining with 1:25 dilution Goat anti-mouse IgG-AF647 (Jackson 115-606-072), and analyzing on Cytek DXP8 flow cytometer. Data from all twenty vials was averaged to determine the mean CAR expression level (46.5%). The CAR copies/cell value for each cell bank was calculated across 10 independent assays in which each sample was diluted in triplicate, and then measured in quadruplicate. Assay accuracy was established by

confirming that the values in three additional assays were within one standard deviation of the mean CAR copies/cell value for each cell bank. The repeatability of the assay was assessed by calculating the Coefficient of Variation (CV) between triplicate dilutions of each cell bank. The CV was ≤ 2.12 % in three independent assays. Intermediate precision was tested by two operators running three independent assays. The CV for each cell bank in this series was ≤ 2.71%. The accurate limit of detection was defined as the lowest amount of CAR copies/cell that was detectable within the lower limits of the standard curve, and was determined to be 0.019 CAR copies/cell. Measurements below this number (that fall outside the range of the standard curve) are detectable with this assay, but are not able to be used for quantification purposes.

Data handling and analysis. Summarized result data for each data type (qPCR, flow cytometry, cytokine analysis) were compiled into an in-house SQLite3 database. For qPCR results, we tracked the mean and standard deviation of the quadruplicate measured CAR copies/cell for each sample, as well as the amount of TNA input into the assay. For flow cytometry, we tracked the cell counts and percentages of cell populations of interest and their parent populations. For cytokine analysis, we tracked the mean and standard deviation of duplicate measured pg/mL values for each cytokine tested for each sample. To aid in correlation of these data with clinical parameters, we also compiled clinical data for each subject and information about each subject's CAR-T product into this database. Correlative analysis was performed in R, extracting data from this SQLite3 database.

SUPPLEMENTAL TABLES AND FIGURES

Supplemental Table 1: Details regarding disease risk for N=30 patients infused

Age	Sex	ECOG	Diag.	Disease stage	Disease Risk	Previous lines of therapy	Response to front-line therapy
36	F	2	ALL	N/A	BCR-ABL -, 46 XX, -3, -7	DFCI, Blina, MAC ALLO, Ino	Primary ref
25	M	1	ALL	N/A	Complex cytogenetics	DFCI, MAC ALLO, Blina, ALL 13-01	Rel < 12mos
64	M	1	ALL	N/A	BCR-ABL+	ALC4, Blina, Ino, RIC ALLO, Ino	Rel < 12 months
75	M	1	ALL	N/A	BCR-ABL+, low hypodiploidy, monosomy 7	TKI/hyper-CVAD, 2nd TKI, Ino, Blina	Primary ref
39	F	0	ALL	N/A	BCR-ABL -, normal karyotype	ALL 13-01, VPCY, MAC ALLO, Blina, Ino	Rel > 12 months
47	F	1	DLBCL	IV	GCB type; IPI 2; BCL2+, BCL6+	RCHOP with HDMTX, GDP, BEAM AUTO, XRT	Primary ref
75	M	0	DLBCL	III	GCB type; IPI 2; MYC+, BCL2+, BCL6+	RCHOP, GDP	Rel > 12 months
73	M	0	DLBCL	IV	GCB type; IPI 2, MYC NA, BCL2 +, BCL6 +	RCHOP, GDP, BEAM AUTO	Rel > 12 months

Supplemental Material

58	M	0	DLBCL	II	GCB type; IPI 1; BCL2+, BCL6+	RCHOP, GDP, ICE	Primary ref
63	M	1	DLBCL	IV	Non-GCB type; IPI 2; BCL2 +	RCHOP, R-GDP	Primary ref
74	F	0	DLBCL	IV	GCB type; IPI 2; BCL2+, BCL6+	RCHOP, XRT	Primary ref
74	M	1	DLBCL	III	IPI 3; MYC+, BCL2+, BCL6+	RCHOP, R-GDP	Primary ref
75	F	1	DLBCL	III	IPI 1; BCL2+, BCL6+	RCHOP, GDP	Primary ref
74	M	1	DLBCL	III	IPI 4; BCL2+	RCHOP, GDP, ICE, XRT	Rel > 12 months
71	M	1	DLBCL	I	ABC type; IPI 2; MYC+, BCL2+, BCL6+	RCHOP, R-CDP	Rel > 12 months
64	F	1	FL	I	IPI 4; BCL6+	RCHOP, GDP	Primary ref
71	M	0	MCL	IV	MIPI 6.5	RCHOP/DHAP, acalabrutinib	Primary ref
67	M	1	MCL	IV	MIPI 6.3	BR, RCHOP/R-DHAP, BEAM AUTO, XRT, ibrutinib	Rel > 12 months
52	M	1	MCL	IV	MIPI 5.7	RCHOP/R-DHAP, BEAM AUTO, TKI, RIC HAPLO	Rel > 12 months
60	M	1	MCL	III	MIPI 5.3	RCHOP, ICE, Gem-R, Benda-R/XRT	Primary ref
72	M	2	MCL	III	MIPI 6.9	Benda-R, Acalabrutinib, RCHOP	Primary ref
65	M	2	MCL	IV	MIPI 6.1	BR, BEAM AUTO, RCHOP, TKI, Venetoclax, GDP	Primary ref
48	M	0	MCL	IV	MIPI 4.3	R-DHAP, Benda-R, R-CEOP, GDP	Primary ref
73	F	1	MCL	III	MIPI 6.5	RCHOP, Benda-R, Ibrutinib, GDP, acalabrutinib, BEAM AUTO	Rel > 12 months
61	M	1	tDLBCL	III	GCB type; IPI 1; BCL2+, BCL6+	RCHOP, GDP, XRT, GDP	Rel > 12 months
67	F	1	tDLBCL	IV	GCB type; IPI 2; BCL2+, BCL6+	R-CVP, XRT, BR, RCHOP, GDP	Primary ref
75	M	1	tDLBCL	II	IPI 0, BCL2+	R-CVP, BR, R, GDP	Rel > 12 months
70	M	1	tDLBCL	IV	GCB type; IPI 1; MYC+, BCL2+, BCL6+	RCHOP, GDP, BEAM AUTO, XRT	Primary ref
29	F	1	PBL	IV	IPI 2	RCHOP/nortezomib, BEAM AUTO, XRT, len, RGDP, RIC ALLO	Primary ref
42	M	0	Richter's	II	CLL IPI 1	FCR, RCHOP, GDP, Ven/ibr, ven, ibr, RIC ALLO	Primary ref

tDLBCL = transformed diffuse large B cell lymphoma; PBL = plasmablastic lymphoma

Supplemental Material

Supplemental Table 2: Antibodies used for multispectral flow cytometry

Fluorochrome	Marker	Clone	Company	Cat #
SparkBlue550	CD14	63D3	Biolegend	367148
PE	TIGIT	MBSA43	FisherSciCan (eBio)	12-9500-42
PE-Dazzle594	CD62L	DREG-56	Biolegend	304842
PerCP	CD8	SK1	Biolegend	344708
PerCP-eFluor710	CD22	4KB128	FisherSciCan (eBio)	46-0229-42
PE-Cy7	CD39	A1	Biolegend	328212
APC	CD137	4B4-1	Biolegend	309810
AF647	CD19	SJ25C1	Biolegend	363040
AF700	CD4	SK3	Biolegend	344622
APC/Fire810	CD45	HI30	Biolegend	304076
BV421	CCR7	G043H7	Biolegend	353208
eF450	CD20	2H7	FisherSciCan (eBio)	48-0209-42
BV480	CD28	CD28.2	BD	566110
BV510	CD45RO	UCHL1	Biolegend	304246
BV570	CD45RA	HI100	Biolegend	304132
BV605	CD27	O323	Biolegend	302830
BV650	HLA-DR	L243	Biolegend	307650
BV711	CD56	HCD56	Biolegend	318336
BV750	CD3	SK7	Biolegend	344846
BV785	PD-1	EH12.2H7	Biolegend	329930

Supplemental Material

Supplemental Figure 1. CAR-T cell and B cell frequencies in participant blood over time as measured by flow cytometry.

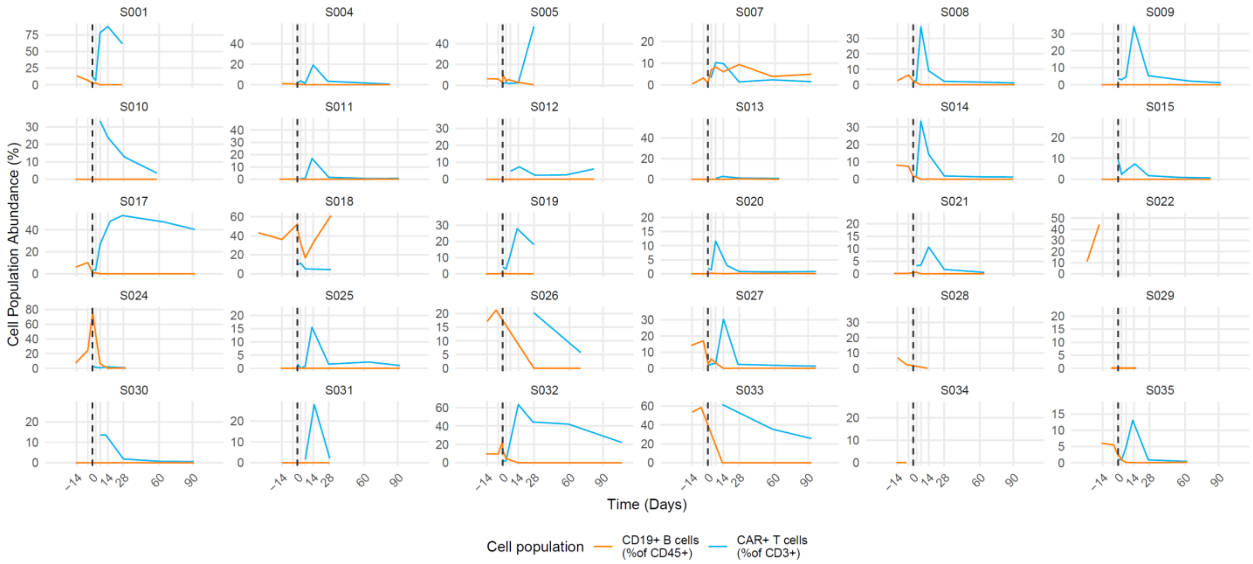

Each panel shows the CAR-T cell (blue) and B-cell (orange) frequencies (y-axis) over time (x-axis). Vertical dashed line at day 0 marks time of CAR-T cell infusion. Generally, post-infusion, B-cell frequencies drop while CAR-T cell frequencies expand.

Supplemental Figure 2. Overview of CAR-T expansion over time in participant blood as measured by qPCR.

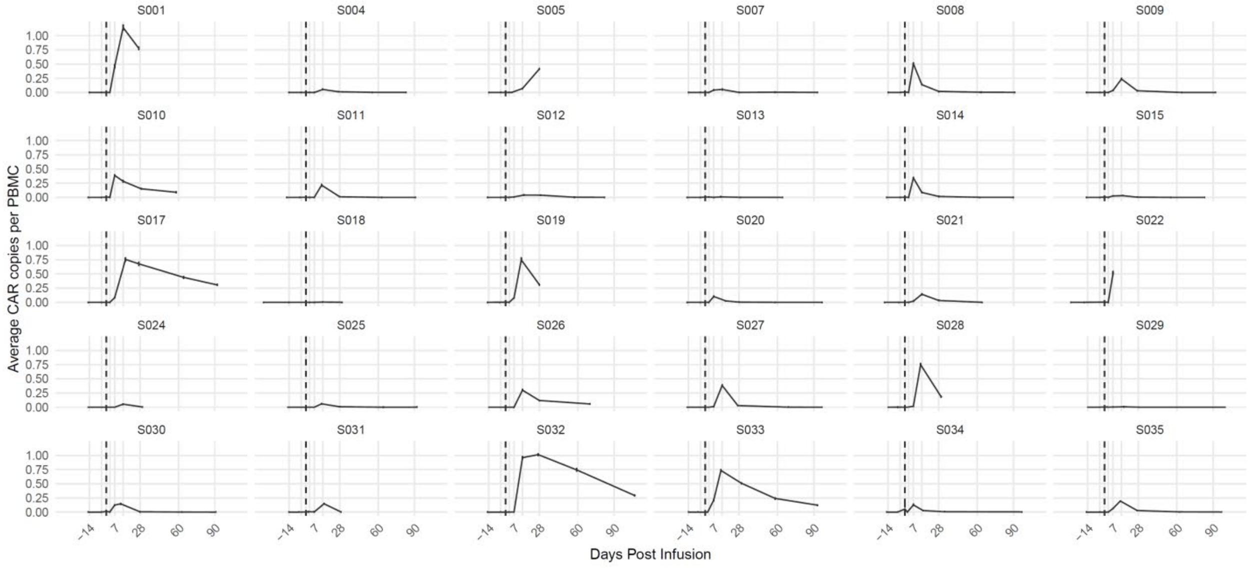

Each panel shows the qPCR-measured CAR genomic copies per PBMC (as a proxy for percent of cells that are CAR+) for a participant at each time point. As expected, CAR genes are not detectable in participant blood at time points prior to CAR-T cell infusion. Generally, CAR levels peak around day 7-14. In some participants, CAR can still be detected in the blood after 90 days.

Supplemental Material

Supplemental Figure 3. Overview of cytokine levels over time in participant blood as measured by Mesoscale.

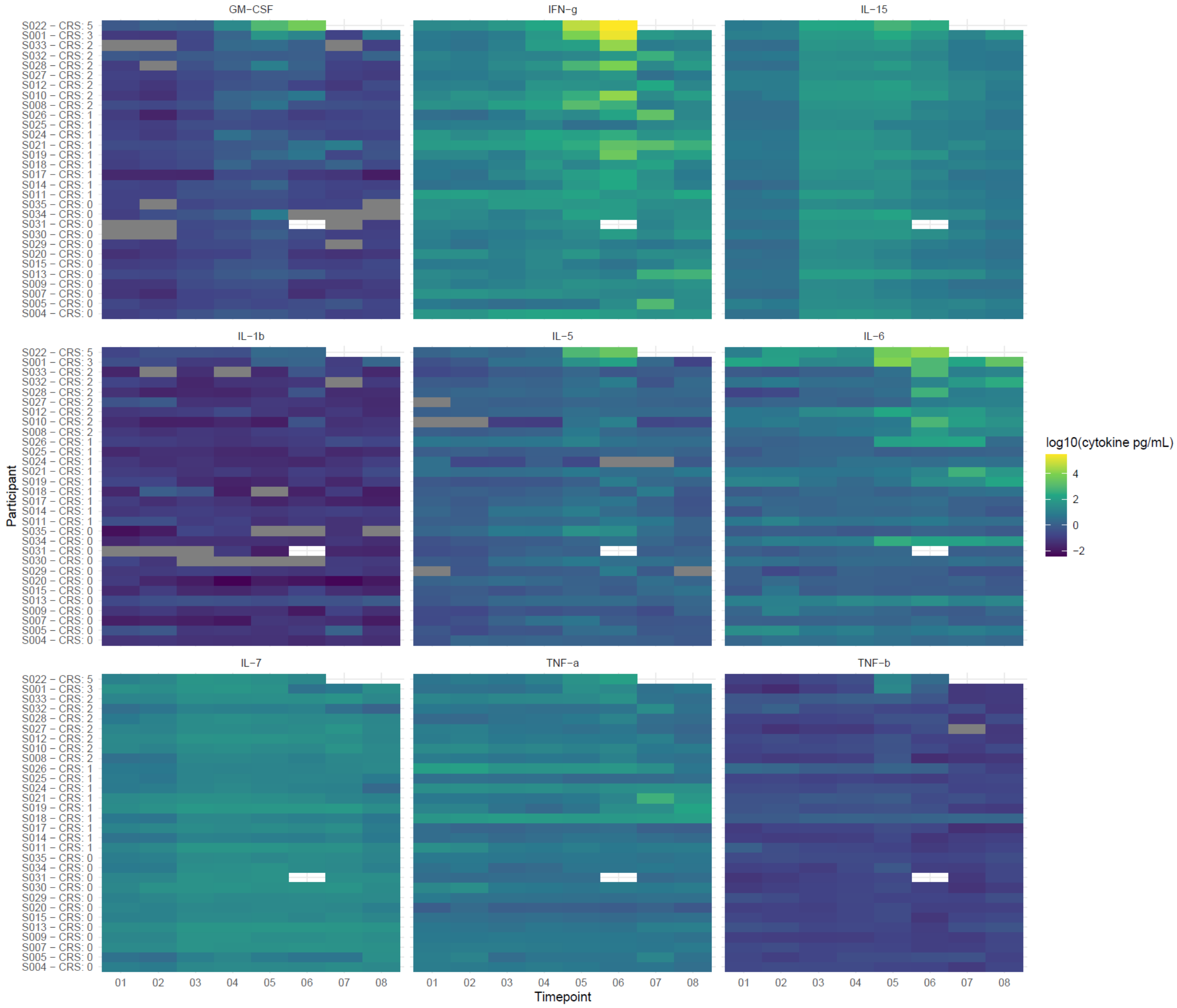

Each panel shows measured cytokine concentrations in participant serum at each time point. Panel headers list the cytokine being assessed, and participants are ordered along the y-axis by decreasing CRS grade (with grade 0 being no CRS). Grey cells denote assay failure, while missing (white) cells denote missing samples. Cytokine concentrations are measured in pg/mL, and displayed after log10 transformation. Participants experiencing CRS grade 3+ show clear increases in GM-CSF, IFN-g and IL-6 at time points 05 and 06 (days 3 and 7). (IL-1a was tested by not displayed on this figure due to a high number of assay failures).
